# The Search for a Schistosomiasis Vaccine: Australia’s Contribution

**DOI:** 10.3390/vaccines9080872

**Published:** 2021-08-06

**Authors:** Donald P. McManus

**Affiliations:** Molecular Parasitology Laboratory, Infectious Diseases Division, QIMR Berghofer Medical Research Institute, Brisbane 4006, Australia; Don.McManus@qimrberghofer.edu.au; Tel.: +61-418-744-006

**Keywords:** *Schistosoma*, schistosomiasis, vaccine, vaccination, Australia, Australian researchers

## Abstract

Schistosomiasis, a neglected tropical disease caused by parasitic flatworms of the genus *Schistosoma*, results in considerable human morbidity in sub-Saharan Africa, in particular, but also parts of the Middle East, South America, and Southeast Asia. The anti-schistosome drug praziquantel is efficacious and safe against the adult parasites of all *Schistosoma* species infecting humans; however, it does not prevent reinfection and the development of drug resistance is a constant concern. The need to develop an effective vaccine is of great importance if the health of many in the developing world is to be improved. Indeed, vaccination, in combination with other public health measures, can provide an invaluable tool to achieve lasting control, leading to schistosomiasis elimination. Australia has played a leading role in schistosomiasis vaccine research over many years and this review presents an overview of some of the significant contributions made by Australian scientists in this important area.

## 1. Introduction

The neglected tropical parasitic disease of schistosomiasis, caused by blood flukes of the genus *Schistosoma,* has long been a scourge of humankind. It results in considerable morbidity, even leading to death, in sub-Saharan Africa, parts of South America, the Middle East and Southeast Asia. It is estimated that 779 million people are at risk of infection, with over 250 million harbouring *Schistosoma* infections, the majority (201.5 million) living in Africa [1,2,3,4]. The 2016 Global Burden of Disease study estimated the global burden due to schistosomiasis at 1.9 million disability-adjusted life years (DALYs) [2], but other reported DALYs have varied substantially, even being as high as 70 million [2].

The schistosome life cycle is complex, involving free-swimming infective larvae (cercariae) which develop in intermediate hosts (fresh-water snails), are released and penetrate the skin of the definitive human host during contact with infested water. In the body, the larvae develop into mature adult male and female worms. These reside in the pelvic (*Schistosoma haematobium*) or mesenteric (*Schistosoma mansoni* and *Schistosoma japonicum*) veins, where the female worms lay eggs, many of which are released to the external environment in urine or stool and hatch in water to release miracidia which seek and penetrate a specific molluscan host and continue the parasite life cycle [2]. Other eggs, however, are entrapped in organs, such as the bladder or liver and surrounding tissues, and cause inflammatory immune responses (including granuloma development), resulting in urogenital, intestinal, or hepato-splenic disease [2]. In regard to treatment, the drug praziquantel (PZQ) is effective against the adult worms of all schistosome species infecting humans, and is safe. Mass drug administration (MDA) with PZQ is the current basis of public health efforts that aim to reduce morbidity due to schistosomiasis. However, PZQ does not prevent reinfection and, although there is no clear confirmation that clinically relevant resistance has developed, the threat of PZQ-resistant schistosomes evolving naturally is a constant concern. It is now generally considered that, as a single-dimension elimination strategy, MDA is unsustainable and that an alternative scenario, involving the deployment of a safe and effective schistosomiasis vaccine, combined with drug treatment and other interventions (e.g., health education; snail control; Water, Sanitation and Hygiene [WASH]), may be the best way forward and this more-versatile approach to control is supported by mathematical modelling [2]. The development of an effective vaccine is of considerable importance to improve the public health of many and in 2016, *Science* ranked the development of a vaccine for schistosomiasis as one of the top 10 needed, based on feasibility and necessity.

Although formidable hurdles remain and we do not yet have available a commercial product, the development and positioning of an effective vaccine within the spectrum of disease control approaches has long been the goal of many researchers globally. In this respect, Australia has been at the masthead of schistosomiasis vaccine research, an area in which it has a rich history. This article provides a chronology of, arguably, some of the more important contributions made by Australian scientists in schistosomiasis vaccine development over the past 45 years.

## 2. Schistosomiasis Vaccine Research in Australia—The Beginning

The doyen and undoubtedly the father of schistosomiasis vaccine research in Australia is Professor Graham F. Mitchell, a highly respected and much honored scientist who has made an indelible mark on the field of parasitology. After graduating in veterinary science at Sydney University, where he was the University gold medalist, Mitchell made pivotal discoveries in basic immunology during the course of his PhD at The Walter and Eliza Hall Institute of Medical Research (WEHI) in Melbourne. With his supervisor, Professor Jacques Millar, who had earlier discovered the immunological function of the thymus gland, he demonstrated that mammalian lymphocytes could be divided into what became known as T cells and B cells, which interact to produce antibodies. Following overseas post-doctoral experience, he returned to Australia and established, at the dawn of the new burgeoning biotechnology revolution, a program in immunoparasitology at WEHI in 1976 [5]. This program became a major component of the global effort to develop new tools, including vaccines, for the control of parasitic diseases, notably falciparum malaria, cutaneous leishmaniasis and schistosomiasis.

Mitchell was instrumental in establishing a long-term and highly productive program on schistosomiasis japonica with Professor Edito Garcia and Dr Wilfred Tiu (who undertook his PhD with Mitchell at WEHI) based at the College of Public Health, University of the Philippines, Manila. A major thrust of this collaborative research involved addressing diverse aspects of the host–parasite relationship and, in particular, to understand the phenomenon and implications of anti-embryonation immunity in schistosomiasis japonica. To do this, the Melbourne–Manila team undertook a number of experiments in mice that provided evidence to support their 1982 hypothesis [6] that granuloma modulation and a decline in chronic disease due to *S. japonicum* could be attributable to antibody-mediated effects on egg maturation and egg viability [7,8,9,10,11].

Schistosomiasis is a classical immunopathologic disease that develops following T cell-dependent immune responses to antigens produced by schistosome eggs entrapped in tissues and organs such as the intestine and liver [2]. Mitchell and Garcia’s rational was that the generation of anti-embryonation immunity, involving destruction of the egg long before it matures to an egg containing a miracidium, would inhibit the production of immunopathologic antigens, resulting in a reduction in the number and size of T cell-dependent granulomas and reduced fibrosis. Indeed, the results of their research showed convincingly that egg-sensitized, *S. japonicum*-infected mice harboured fewer mature eggs and more dead eggs in the livers and intestines compared with similarly infected but unsensitized control mice. Furthermore, the egg-sensitized animals had fewer and smaller granulomas around eggs in the liver, smaller spleens and reduced portal pressure. Overall, the outcomes of these mouse experiments strongly supported their earlier premise that it should be possible to prevent or restrict serious hepatosplenic disease by inducing anti-embryonation immunity through vaccination.

Anti-embryonation immunity has potential implications as well for transmission dynamics in schistosomiasis-endemic communities as fewer mature eggs would likely be produced and/or their access into the intestinal lumen would be reduced, resulting in smaller numbers of hatched miracidia in the external environment to infect the *Oncomelania* intemediate host snails. Similarly, Mitchell and his team advanced the additional possibility that anti-egg-induced immunity would result in anti-fecundity effects perturbing oviposition, and that female miracidia within eggs of chronically infected individuals may be more prone to anti-embryonation immunity damage than male miracidia, again with important repercussions for schistosomiasis transmission and the epidemiological consequences resulting. Overall, the anti-embryonation immunity studies by Mitchell and his collaborators provided considerable encouragement and pointed the way ahead to future immune intervention studies aimed at developing anti-infection, anti-disease and transmission-blocking vaccines for schistosomiasis, a path that, as will be discussed, many researchers subsequently followed.

## 3. The GST Story

In the search to identify schistosome antigens capable of inducing host-protective immunity and thus having potential as a molecular vaccine against schistosomiasis, Mitchell’s group investigated the basis of the resistance of inbred 129/J mice raised at his institute (designated WEHI 129/J) to infection with *S. japonicum* [12]. The research showed that approximately 50% of exposed WEHI 129/J mice were resistant in that they harboured no adult worms; in contrast, all mice of a susceptible strain such as BALB/c became infected following challenge with Philippines *S. japonicum* cercariae [12]. A similar phenomenon occurred with *S. mansoni* in that conventionally maintained 129/J mice bred at WEHI were also resistant to this schistosome species [12]. In further experiments, schistosome-exposed WEHI 129/J mice displayed serum antibody specificities that were different from those found in sera from infected BALB/c mice, and, in particular, these animals were shown to be high responders to a *S. japonicum* adult worm antigen of Mr 26,000 (termed Sj26) [12].

Then followed some very elegant molecular and biochemical studies by the team at WEHI showing that: (a) clones, corresponding to the Mr 26,000 adult worm antigen, were identified as β-galactosidase fusion proteins in a phage Xgtll-amp3 expression library of *S. japonicum* adult worm mRNA using affinity-purified antibodies eluted from the 20–30 kDa region of electrophoresed *S. japonicum* adult worm antigens in Western blots; (b) the deduced amino acid sequence of the cDNAs had striking homology with published sequences of mammalian glutathione *S*-transferases (GSTs); (c) adult *S. japonicum* worms had substantial levels of GST activity and that Sj26 was able to bind glutathione, a property that was harnessed in a novel pGEX expression system (developed by Donald Smith at WEHI), and subsequently made available commercially as a ‘Glutagene’ kit; this enables soluble fusion proteins consisting of Sj26 and other sequences to be purified from *E. coli* in a single step on glutathione-conjugated agarose columns; (d) S. *japonicum* and S. *mansoni* each have at least two GST isoenzymes of 26 and 28 kDa (Sj26, Sj28, Sm26 and Sm28) with the amino acid sequences indicating that the 26 and 28 kDa GSTs are distinct from one another; Sj26 and Sm26 having homology to class µ mammalian GSTs and Sj28 and Sm28 being similar to class α GSTs [13,14].

The GSTs were considered attractive vaccine candidates due to several biological features. Expressed abundantly in schistosomes, Mitchell hypothesised that they help the worms survive in their mammalian hosts by protecting them from: various toxins circulating in blood; against membrane damage due to products of lipid peroxidation generated by aggressive attack by immune-effector cells at the parasite surface; and/or by increasing the solubility of haematin in the schistosome gut thereby helping to reduce “constipation” of worms [12]. However, despite the very elegant and informative studies undertaken by Mitchell and his group, Sj26 proved not to induce consistent (if any) host-protective immunity to Philippines *S. japonicum* in a number of mouse strains at WEHI. This raised doubts whether satisfactory levels of resistance to *S. japonicum* were attainable by vaccination with Sj26 alone. Indeed, a large series of subsequent experiments by Mitchell and his team failed to induce resistance in mice to *S. japonicum* or *S. mansoni* in a number of different vaccine regimens including exposure of animals to crude schistosome antigens plus cloned GSTs [15].

Furthermore, it had become clear that liver and portal system peculiarities, possibly due to nutritional factors, such as hypervitaminosis A or an infectious cause, contributed to the apparent resistance of 129/J and some C57BL/6 mice maintained at WEHI with variable numbers of adult worms establishing after cercarial challenge [16]. Similar peculiarities with C57BL/6 mice were reported subsequently by McManus’s Australian team, who also showed that the complete recombinant Sj28 protein failed to protect mice consistently against subsequent challenge [17]. Along with Chinese collaborators, however, McManus reported solid anti-fecundity immunity generated with the 26 kDa GST (SjcGST26) of Chinese strain *S. japonicum* in mice, pigs, and, importantly, water buffaloes [17] which, as will be discussed further later, can act as major reservoirs of schistosomiasis japonica [17]. Following a separate line of research to that of Mitchell, Andre Capron and colleagues in Lille, France demonstrated that immunization of several host species with the 28 kDa GST of *S. mansoni* (SmGST28) followed by experimental infection led to a reduction in worm burden and/or a significant decrease in parasite fecundity, establishing this antigen as a promising vaccine candidate, thereby reinforcing the premise that anti-fecundity immunity could be incorporated as a relevant component of vaccine strategies against schistosomiasis [18,19]. Due to clinical and epidemiological reasons, priority for the subsequent passage to human clinical trials was given by Capron and his group to the severe urogenital form of human schistosomiasis due to *S. haematobium.* Extensive additional research by the French team led to subsequent clinical trials of the 28 kDa GST of *S. haematobium* (rSh28GST) (Bilhvax), the homologue of SmGST28. Bilhvax is one of only three *Schistosoma* vaccines to have entered human clinical trials [20,21,22], the other two being Sm14 (14 kDa cytoplasmic fatty acid binding protein) and Sm-TSP-2, a 9 kDa tetraspanin integral membrane protein discovered, as now discussed, by Australian scientists.

## 4. The Sm-tetraspanin-2 (Sm-TSP-2) Vaccine

Surface and secreted proteins are key components of schistosome worms directing the basic physiological necessities of a parasitic existence. They represent clear vaccine targets being intimately exposed to host tissues during penetration, migration and feeding, and they are involved in evading the host immune response [23,24,25]. Alex Loukas and his team in Brisbane used the novel approach of Signal Sequence Trap (SST) to select *S. mansoni* cDNAs encoding secreted and surface proteins with N-terminal signal peptides; as a result, two cDNAs of especial interest were revealed: *Sm-tsp-1* and *Sm-tsp-2* [25,26]. These mRNAs encode two major *S. mansoni* tetraspanins (TSPs) (Sm-TSP-1 and Sm-TSP-2), four-transmembrane-domain proteins homologous to surface receptors on B and T cells, which likely play important structural roles in tegument biogenesis and integrity [27]. Loukas et al. [26] raised antibodies in mice to the large extracellular loop 2 (major ligand-binding domain) of Sm-TSP-1 and Sm-TSP-2 as soluble recombinant fusion proteins with *E. coli* thioredoxin and showed that both molecules are exposed on the tegumental surface of adult *S. mansoni* worms. The group further showed that recombinant Sm-TSP-2, but not Sm-TSP-1, was recognized strongly by IgG1 and IgG3 (but, importantly, not IgE) antibodies in sera from naturally resistant individuals but not by IgG from unexposed or chronically infected individuals. In addition, Sm-TSP-2 generated a level of protection in the mouse vaccine model in excess of the 40% benchmark set earlier by the World Health Organization for progression of schistosome vaccine antigens into clinical trials.

Accordingly, coupled with the level of protective efficacy generated with its selective recognition by naturally resistant people, Sm-TSP-2 was selected for further process development and clinical trialing as a prime schistosomiasis vaccine candidate [28,29]. The vaccine is currently being progressed as a 9 kDa recombinant Sm-TSP-2/Alhydrogel^®^ vaccine combined with glucopyranosyl lipid A aqueous formulation (GLA-AF) as adjuvant by the Sabin Vaccine Institute Product Development Partnership in the USA. Sm-TSP-2 has been scaled up in yeast using the Pichia pink system, it has undergone extensive toxicology studies, and it exhibited good pre-clinical results; several clinical trials with the vaccine have been completed are progressing in non-endemic and endemic communities [29]. These include an initial phase-1 trial at the Baylor College of Medicine, Houston, TX, USA and a phase-1b study in Brazil to assess the vaccine’s immunogenicity and safety in a group of healthy adults who may have been previously exposed to schistosomiasis; further trialing of Sm-TSP-2 are planned for Uganda (https://clinicaltrials.gov/ct2/show/{“type”:”clinicaltrial”,”attrs”:{“text”:”NCT03910972”,”term_id”:”NCT03910972”}}NCT03910972) (accessed on 20 July 2021) [29].

## 5. Transmission-Blocking Vaccines—The Background

The zoonotic nature of schistosomiasis japonica complicates the control and transmission of *S. japonicum* in the Philippines and the People’s Republic of China; cattle and water buffalo are the most important reservoir hosts with up to 90% of egg contamination estimated as originating from this source [30,31,32]. This is not unduly surprising given the daily fecal output from a water buffalo (∼25 kg) has been estimated to be at least 100 times that produced by a human individual (250 g) [33]. Australian and Chinese scientists were among the first to advocate the vaccination of reservoir host animals, particularly bovines, to assist in long-term prevention of human (and animal) *S. japonicum* infection, a concept reinforced by mathematical modelling [34]. The possibility that this strategy could pay off gained backing from Australian/Chinese studies showing that the animal-snail-human transmission cycle is more prominent than the human-snail-human one in sustaining the infection [35].

In further support, a praziquantel (PZQ)-based intervention study (1998 to 2003) [36] around Poyang Lake in Jiangxi Province led by Australian and Chinese scientists showed experimentally that bovines are major reservoirs for human *S. japonicum* infection. The trial demonstrated that the drug treatment of bovines impacted human infection rates by demonstrating a greater reduction in human incidence in an intervention village (all the humans and bovines received PZQ treatment) compared with a control village (all the humans only received PZQ treatment). Mathematical modeling supported this conclusion and predicted that water buffaloes were responsible for approximately 75% of human transmission in this setting [36].

A similar but more rigorous PZQ intervention trial (2004 to 2007) was then undertaken in bovines by the same team of Australians and Chinese [37,38]. The trial had increased power involving a cluster-randomized design, and had the further aim of providing general applicability to the lakes and marshlands of southern China. The cluster-randomized trial comprised four matched village pairs in Hunan and Jiangxi Provinces, with one village within each pair randomly selected as the control group (human PZQ treatment only) with the other designated as the intervention group (human and bovine PZQ treatment). A sentinel human cohort was selected from each village for the duration of this study to monitor new infections. The results indicated that a combination of human and bovine chemotherapy had a greater effect on human incidence than human treatment alone, an outcome that was supported by Poisson regression analyses. This trial provided further proof that the incidence of human *S. japonicum* infection could be decreased through the reduction in infection rates in water buffaloes, thereby reinforcing the development and deployment of a transmission-blocking vaccine. Whereas a reduction in worm numbers is the gold standard for anti-schistosome vaccine development, as earlier indicated, a vaccine targeting parasite fecundity and egg viability is also applicable, given the eggs are responsible for both the pathology and transmission of schistosomiasis.

A transmission-blocking vaccine would be particularly useful in areas deemed unsuitable for the replacement of bovines by mechanized farming, one of the interventions featuring in the successful integrated schistosomiasis control strategy in China [39]. Indeed, the application of an animal-based transmission-blocking vaccine as a component of a suite of integrated control measures for schistosomiasis fits well with the One Health concept synergizing animal and human health.

## 6. Transmission-Blocking Vaccines—Candidate Antigens

Extensive laboratory and field research, undertaken by Australian and international scientists, has identified a set of well-defined *S. japonicum* molecules that may be involved in inducing protective immune responses, with a view to developing them further as viable transmission-blocking vaccines targeting the prevention of schistosome infection, the reduction in parasite fecundity, or both. Many of the lead *S. japonicum* vaccine candidates are enzymes, muscle components and membrane proteins; further details of their characteristics and vaccine efficacies can be found in recent reviews, a number of which were authored/co-authored by Australian researchers [40,41,42,43,44,45,46]. The candidate antigens that have received most attention as potential components of a transmission-blocking vaccine against schistosomiasis japonica have been glutathione S-transferases, paramyosin, triose-phosphate isomerase and insulin receptor, now briefly described.

### 6.1. Sj-26GST

As earlier discussed, extensive initial research showed the most repeatable host protection produced against the GSTs of all schistosome species was the significant reduction in female worm fecundity, resulting in decreased egg output and viability and, consequently, both a decrease in egg-induced pathology and reduced transmission [47]. Working with Chinese colleagues, McManus demonstrated protective efficacy (the characteristic anti-fecundity effect) in mice, water buffaloes, cattle and pigs following vaccination with the 26kDa GST isoform (Sj-26GST) [41,42,48,49,50,51]. Furthermore, field-testing of the Sj26GST by the same team showed the protective effect against *S. japonicum* was maintained in bovines for at least 12 months post-vaccination [51]. Despite the early success, it is unfortunate that there have been no further trials assessing the efficacy of the Sj-26GST vaccine in animal livestock likely due to funding restrictions as these large animal trials are expensive and logistically challenging.

### 6.2. Sj-97

Paramyosin (Sj-97) is a large (97 kDa) coiled-coil myofibrillar protein; in schistosomes it is located on the surface tegument of lung stage schistosomula, in the muscles of larvae and adult worms, and in the secretory glands of cercariae. Attention was first paid to paramyosin as a vaccine candidate following highly encouraging results of vaccine/challenge experiments in mice targeting *S. mansoni* [52]. Then followed trials coordinated by Australian and international researchers in mice, sheep, pigs and water buffaloes with native or recombinant full-length and recombinant fragments of *S. japonicum* paramyosin (rSj-97), resulting in significant, albeit partial protection being generated [53,54,55,56]. Following the development of a robust method to express and purify rSj-97, and incorporating the adjuvant Montanide ISA 206, the paramyosin-based vaccine was shown by Philippines and USA researchers to be safe, well tolerated and highly immunogenic in water buffaloes both in a highly endemic area for *S. japonicum* in the Philippines and in the People’s Republic of China [56].

### 6.3. SjTPI

Triose-phosphate isomerase (TPI) is present in most cells of adult schistosomes, and has been shown to be localised on the surface membranes of the newly transformed schistosomulum, the stage in the mammalian host that is considered the most likely target of an anti-schistosome vaccine [41]. TPI has been assessed for vaccine efficacy in mice, bovines and pigs [41,42], and Australian scientists were involved in testing a plasmid-generated (Chinese strain of *S. japonicum*) DNA vaccine (SjCTPI- heat shock protein (hsp)-70), co-administered with IL-12 as adjuvant; this induced very good protective efficacy against *S. japonicum* in Chinese water buffaloes [57]. The vaccine was subsequently field-tested in bovines against natural *S. japonicum* challenge in China’s Hunan Province in a double-blinded, phase-3 cluster randomized controlled trial (RCT) [58]. The RCT assessed the impact on schistosomiasis japonica transmission of a multi-component integrated control strategy that included the SjCTPI vaccine, the use of the chemical molluscicide niclosamide against the intermediate snail host (*Oncomelania hupensis*) of *S. japonicum* and human praziquantel treatment. The vaccination regimen involved heterologous (DNA-protein) prime-boost delivery of the vaccine whereby bovines had the SjTPI-hsp-70 DNA vaccine as a prime, and were given a vaccination booster with recombinant SjTPI protein using VacSIM (vaccine self-assembling immune matrix) delivery without adjuvant [58].

Some outcomes of this large highly challenging RCT were positive but the overall impact of the SjTPI vaccine in the prevention of human infection was inconclusive mainly due as a result of the removal, sacrifice or treatment of bovines over the trial duration by the Chinese authorities [59]. Indeed, the majority of livestock animals in the previously high schistosomiasis-endemic Poyang and Dongting Lake areas have been removed being replaced by mechanized tractors as an approach that may help provide the final component required for elimination of the disease [59]. A similar RCT of bovine vaccination by Australian, American and Philippines scientists, using a multi-factorial design incorporating the SjTPI vaccine, has been completed in the Philippines (Allen G. Ross, personal communication), although the results of the trial have yet to be released.

## 7. Insulin Receptors (SjIRs)

Adult schistosome worms absorb large quantities of glucose from their mammalian hosts, consuming their dry weight of this sugar every 5 h [60]. The glucose is critical as it provides energy in the form of ATP for a number of worm functions including growth, development, pairing, maturation and egg production. Interrupting or blocking glucose uptake presents a logical approach to vaccine development as it would lead to decreased energy synthesis and result in starvation of the parasites, thereby affecting these vital cellular processes. Australian scientists demonstrated that *S. japonicum* possesses two types of insulin receptors (SjIRs) (SjIR1 and SjIR2) which modulate glucose uptake from host blood into the worm by binding to mammalian host insulin, activating the parasite’s insulin pathway, a process that is pivotal for the process (60). SjIR2 is localised to the vitelline tissue of females and in the parenchyma of males whereas SjIR1 is positioned on the internal epithelium and tegument basal membrane of adult worms [60].

In a series of subsequent vaccine/challenge trials in mice, the Australian group showed that recombinant fusion proteins of the ligand domains of SjIR1 and SjIR2 (rSjLD1 and rSjLD 2) conferred solid protection (depressed female growth, reduced intestinal granuloma density and reduced fecal egg production) in mice against challenge infection with *S. japonicum* [61,62,63]. Furthermore, the same team showed that RNA interference (RNAi) knock down of the two SjIRs led to their reduced expression in worms and that this was coupled with reduced levels of transcription of downstream insulin pathway genes that are associated with glucose metabolism and schistosome fecundity [62]. This emphasised the promise of the two *S. japonicum* insulin receptors as vaccine candidates but they will need to be rigorously field-tested in the future to determine their full potential.

As with the African schistosomes, the development of an efficacious *S. japonicum* vaccine has proven a challenge but the deployment of a transmission-blocking vaccine, in tandem with other public health measures (e.g., effective human and animal treatment, snail control, improved water, sanitation and hygiene, and health education), for the prevention of *S. japonicum* will be pivotal if the goal of elimination is to be achievAustralian input into schistosome genomics, post-genomics and novel vaccine antigen discovery

The vaccinology field in general has recently undergone rapid evolution, driven by the current COVID-19 pandemic and by recent advances in our understanding of immunology, genomics, proteomics and other omics technologies forging new frontiers in vaccine capabilities. These new technologies will be equally important for the future development and deployment of schistosomiasis vaccines as, notwithstanding recent encouraging progress, especially in the clinical testing of lead molecules, the vaccine antigens currently identified may still not provide the required level of immunological protective potency, so it is vital that the search for novel target candidates continues both in Australia and internationally.

There is no doubt that critical to many of the recent (and future) advances in schistosome antigen discovery have been the publication of the nuclear genomes for *S. japonicum, S. mansoni, S. haematobium* and other schistosome species, a research area in which Australian scientists have made valuable contributions [64,65,66,67,68,69,70]. Australian researchers have also contributed substantially in other related omics areas using a range of research tools that provide a vantage point promising rapid progress in schistosomiasis vaccinology [71,72]. In particular, Australian researchers are exploiting the treasure trove of recently acquired genomics information by utilising cutting-edge techniques in transcriptomics [73,74,75,76,77,78,79,80], proteomics [81,82,83,84,85,86,87,88,89,90,91], immunomics [28,92,93,94,95,96,97,98,99,100,101,102,103], DNA microarray profiling [104,105,106,107,108,109,110,111], glycomics [72,112] and exosomics [89,113,114,115,116], to provide a road-map for revealing future novel schistosome vaccine antigens.

Schistosomes are furnished with small RNA interference machinery and gene manipulation has been used to target different life cycle stages of the three most clinically relevant schistosome species; the approach may help guide the development of novel anti-schistosome interventions including vaccines [71,72]. Indeed, Australian, along with international scientists, have pioneered the application of RNA interference (RNAi) to help decode key biological functions in schistosomes, such as those involved in the maintenance of tegumental integrity, apoptosis and self-renewal, nutrient uptake, reproductive biology and fecundity, gene regulation and immune modulation mechanisms; importantly, RNAi can reveal pivotal genes encoding proteins essential for parasite survival that can be the targets of vaccine orchestrated immunological attack [71,117,118,119].

Although genome editing for functional studies of schistosomes and other helminth parasites is in its infancy, this novel procedure using clustered regularly interspaced short palindromic repeats (CRISPR)/CRISPR-associated protein 9 (Cas9)) has captured considerable recent attention as a powerful procedure to precisely target and then deactivate the genetic information a cell needs to produce a given protein [120]. One application of this technology for the study of schistosomes was recently reported by an Australian team who reported successful gene knock-in (KI) of acetylcholinesterase into the genome of the egg of *S. mansoni* by combining CRISPR/Cas9 with single-stranded oligodeoxynucleotides (ssODNs) [121]. In tandem with two other recent reports [122,123], this pivotal approach can provide a blueprint for editing other schistosome genes encoding specific protein-encoding genes implicated in the disease process associated with schistosomiasis, and as a vehicle to identify novel anti-schistosome vaccine candidates.

Another fledgling area where Australian vaccinologists can make a mark is the establishment of schistosome stem cell lines to advance schistosomiasis vaccine research [124]. The availability of such self-renewable resources can provide a new platform to study stem cell behavior and regulation, and to undertake studies on important areas of schistosome biology, reproductive development and survival. As a result, such research can provide new avenues for unravelling individual gene function and to optimize human blood fluke genome-editing processes, which may lead to the design of novel interventions, including vaccines, for schistosomiasis. Conclusions and the future

Schistosomiasis is an ancient human affliction and this acute and chronic disease has considerable public health consequences globally, especially in the most marginalised and poorest communities. The potential for the development of drug resistance and the incomplete efficacy of praziquantel emphasise the need to continue research into developing effective and inexpensive schistosomiasis vaccines (and drugs). Despite much recent progress and post-genomic research developments, currently no schistosomiasis vaccines have been accepted for public use. The complexity of the schistosome life cycle and the capacity of these bloodflukes to evade host immunity add to the challenge of effective vaccine design, development and deployment. Major advances in genomics and post-genomics technologies have aided new antigen discovery but the schistosomes have largely resisted successful vaccine development efforts, and it is possible that the important immunological targets remain to be identified so research in this important area needs to be continued if not expanded.

## 8. Conclusions

Although in some areas schistosomiasis control will be achievable via current MDA programs, global control and eventual elimination will require a multiple intervention approach involving suitably deployed vaccines integrated with treatment, improved water, sanitation and hygiene, snail control, health education and communication, accurate diagnostics, and surveillance-response systems that are tailored appropriately to discrete social-ecological settings [2]. Akin to many other neglected tropical diseases, schistosomiasis is a disease of poverty, and strong local and international involvement and backing will be required to provide the support for vaccine development and the deployment of the other required multi-faceted interventions if this ancient human scourge is to be consigned to history. Taking into account the extent of consolidated research to generate anti-schistosome vaccines, there is guarded optimism that these endeavours will prevail with Australian scientists, their collaborators and a cohort of international researchers recognized as having played a major role when this successful outcome is realised.
